# Review of Toxic Epidermal Necrolysis

**DOI:** 10.3390/ijms17122135

**Published:** 2016-12-18

**Authors:** Victoria Harris, Christopher Jackson, Alan Cooper

**Affiliations:** 1 Sydney Medical School—Northern, University of Sydney, 2065 Sydney, Australia; 2 Sutton Arthritis Research Laboratory, Kolling Institute, University of Sydney, 2065 Sydney, Australia; chris.jackson@sydney.edu.au; 3 Dermatology Department, Royal North Shore Hospital, 2065 Sydney, Australia; alanjco@tpg.com.au

**Keywords:** inflammatory dermatoses, toxic epidermal necrolysis, drug reaction

## Abstract

Toxic epidermal necrolysis (TEN) is a rare but life threatening mucocutaneous reaction to drugs or their metabolites. It is characterised by widespread keratinocyte apoptosis and sloughing of the skin, erosions of the mucous membranes, painful blistering, and severe systemic disturbance. The pathophysiology of TEN is incompletely understood. Historically, it has been regarded as a drug-induced immune reaction initiated by cytotoxic lymphocytes via a human leukocyte antigen (HLA)-restricted pathway. Several mediators have been identified as contributors to the cell death seen in TEN, including; granulysin, soluble Fas ligand, perforin/granzyme, tumour necrosis factor-α (TNF-α), and TNF-related apoptosis-inducing ligand. Currently, granulysin is accepted as the most important mediator of T cell proliferation. There is uncertainty around the accepted management of TEN. The lack of definitive management guidelines for TEN is explained in part by the rarity of the disease and its high mortality rate, which makes it difficult to conduct randomised control trials on emerging therapies. Developments have been made in pharmacogenomics, with numerous HLA alleles identified; however, these have largely been ethnically specific. These associations have translated into screening recommendations for Han Chinese.

## 1. Introduction

Toxic epidermal necrolysis (TEN) is characterised by widespread, full-thickness necrosis of the epidermis, most likely caused by drugs or their metabolites. The epidermal shedding leaves an exposed and inflamed dermis and the patient susceptible to infection, large fluid shifts, loss of thermoregulation, and electrolyte imbalance. Consequently, there is a high mortality rate of 30% and considerable long-term morbidity in TEN [1]. Lyell first reported four cases of acute onset of a blistering eruption, similar to scalding and associated with systemic upset [2]. The pathology of TEN was also first described at this time as circulating toxins that specifically target the epidermis with resultant necrosis. One of these cases was later redefined as staphylococcal scalded skin syndrome. Based on these early descriptions of the lesions, it was initially thought that TEN and erythema multiforme were on the same spectrum as a single drug hypersensitivity disease. Today, the extent of both diseases is better understood, and erythema multiforme is widely accepted as a separate disease with differences in severity, demographic features, and is associated more with infection (mycoplasma pneumonia and herpes simplex virus) [3]. Studies have also shown via comparison of the histology and immunochemistry that the pathogenesis of the two diseases is distinctly different [4].

TEN exists on the same clinical spectrum as Stevens–Johnson Syndrome (SJS), as a more severe variant. SJS was first described in 1922 by Stevens and Johnson [5], who reported two paediatric cases of widespread generalised eruption, persistent fever, buccal mucosa inflammation, and conjunctival inflammation. The classification of SJS and TEN is along a continuum of extent of body surface affected by epidermolysis [6]:

1. SJS involves less than 10% of total body surface area
2. SJS/TEN involves between 10% and 30% body surface area
3. TEN involves more than 30% body surface area

Toxic epidermal necrolysis has the hallmark histological feature of full-thickness keratinocyte necrosis. Two skin biopsies should be taken from skin adjacent to a blister for routine histopathology. Early lesions show apoptotic keratinocytes scattered in the basal layer of the epidermis, and in established lesions, full-thickness epidermal necrosis and subepidermal bullae may be seen. A second biopsy from periblister skin should be sent for direct immunofluorescence in order to exclude other immunological dermatoses [7]. For example, drug-induced linear IgA bullous dermatosis can produce a bullous eruption that may appear clinically similar to the early stages of TEN. Immunofluoresence will show linear deposition of IgA at the basement membrane.

## 2. Epidemiology

The estimated incidence of TEN and overlap of SJS/TEN (based on European epidemiological studies) range from an annual risk between 0.93 per million and 1.89 per million per year [8,9]. Based on a large European registry study, SJS is more common than TEN, and both SJS and TEN are more common in women than in men [10]. The incidence of SJS/TEN is also considerably higher in the HIV-positive population, and has been estimated at 1–2 per 1000 individuals in the population [11]. This could be due to the increased ingestion of drugs used in these patients, the immunodeficiency itself, and/or the associated infections experienced in these patients.

## 3. Etiology

Drugs are the leading reported cause of TEN, with the risk of a hypersensitivity reaction mainly in the first few weeks of the drug ingestion [12]. A large multi-national case-control study conducted in Europe identified strong associations between SJS/TEN and several drugs, including anti-infectives sulphonamides, allopurinol, carbamazepine, phenobarbital, nevirapine, lamotrigine, phenytoin, and oxicam-non steroidal anti-inflammatory drugs (NSAIDs) [13]. TEN has also been reported to occur following measles-mumps rubella vaccination and *mycoplasma pneumonia* infection—particularly in children [14,15].

## 4. Pathophysiology

### 4.1. Mechanism of Cell Death

The widespread keratinocyte cell death seen in TEN has been attributed to apoptosis or programmed cell death as opposed to necrosis. Electron microscopy examination of lesional skin biopsies from patients with TEN demonstrate characteristic ladder pattern of DNA cleavage that is the biochemical hallmark of apoptosis [16]. Understanding of the pathogenesis of TEN came from studies examining the blister fluid of patients with TEN, where an abundance of CD8 T lymphocytes and Natural Killer (NK) cells were found [17,18]. Thus, TEN appears to be a cell-mediated cytotoxic reaction against keratinocytes that leads to keratinocyte apoptosis. This was later confirmed in a study that extracted the CD8 T cells from patients with TEN and demonstrated their cytotoxic capability of keratinocyte lysis in a major histocompatibility complex (MHC)-I restricted, drug specific manner [19]. Drugs can stimulate the immune system by directly binding to the MHC-I and the T-cell receptor, which results in the clonal expansion of a specific population of cytotoxic T cells. These cytotoxic T cells go on to cause keratinocyte death, both directly and indirectly via recruitment of cells that release soluble death mediators.

### 4.2. Mediators of Keratinocyte Apoptosis

Drug-specific cytotoxic T cells and NK cells may not be the sole effector mechanism of the keratinocyte death, and their action may be amplified by the production of multiple cell-death mediators, altered anti-apoptotic pathways, and altered or defective regulation of drug-specific immune reactions [20]. Various cytotoxic proteins and cytokines have been implicated as mediators of apoptosis in TEN, including granulysin, Fas–Fas ligand interaction, tumour necrosis factor-α (TNF-α), TNF-related apoptosis-inducing ligand (TRAIL), and perforin-granzyme B [21].

#### 4.2.1. Granulysin

A pivotal study by Chung et al. identified granulysin as the main cell death mediator involved in TEN [22]. Granulysin is a cytolytic protein produced and secreted by cytotoxic T lymphocytes (CTLs) and NK cells. The study involved gene expression profiling of cells from five patients with TEN and identified granulysin as the most highly expressed cytotoxic molecule. The blister content of these patients demonstrated cytotoxicity when incubated with keratinocytes, and dampening of this effect was noted with depletion of granulysin; i.e., the levels of granulysin from patient blister fluid correlated with disease severity. In addition, the injection of granulysin from TEN patient blisters into mice skin induced dose-dependent blistering and cell death.

#### 4.2.2. Death Receptor (DR)—Fas Ligand/TNF-α

The Fas–Fas ligand pathway is another proposed pathway for the necrosis and widespread cytotoxic T lymphocyte-mediated apoptosis in TEN. Viard et al. showed evidence that the massive apoptosis in TEN is mediated through activation of the “death receptor” (DR), Fas [23]. Upon recognition of Fas ligand (FasL), Fas undergoes conformational changes in its cytoplasmic death domain that causes recruitment of an “adaptor protein” called Fas-associated death domain protein (FADD). This leads to a caspase cascade where the protease dismantles the cell internally in an orderly fashion. Viard et al. showed that skin biopsies of patients with TEN had dense keratinocyte localisation of FasL, and the serum of these patients had elevated levels of soluble FasL (sFasL).

A subsequent study by Abe et al. [24] was unable to duplicate the findings of Viard. While they confirmed consistently elevated levels of sFasL in the serum, the biopsies of patients’ skin did not show FasL on the surface of keratinocytes. They concluded the elevated sFasL was not from keratinocytes, but from the peripheral blood mononuclear cells. Therefore, while FasL may not be the primary mediator, it has been established that sFasL is significantly increased before the detachment of skin in TEN, and may play a role as a marker of disease for diagnostic purposes at initial presentation [25].

Other DRs such as TNF-R1, DR4 and 5, and their ligands TNF-α and TRAIL may also play a role in the pathogenesis of TEN. However, therapeutic administration of TNF antagonists for TEN remains cautionary because of the known anti-apoptotic properties of TNF-α [26].

### 4.3. Initiation of Apoptosis

The mechanism by which CTLs are activated to cause the widespread keratinocyte death and the aforementioned mediator release remains contentious. Two established theories of CTL activation include the classical pro-hapten model, whereby drugs are metabolised prior to presentation by human leukocyte antigen (HLA) to T cell receptor, and the pharmacological interaction (p-i) concept, whereby the drug does not need to be metabolised and can non-covalently bind directly to the MHC molecule and T-cell receptor without being metabolized [27]. A more recent theory on the mechanism of CTL activation is the “altered self-repertoire model” [20]. In this model, it is proposed that the drug binds non-covalently with the MHC complex, resulting in an alteration of the binding cleft and the self-peptide. This new self-peptide then goes on to activate CTL.

## 5. Genetics

The increase in genome-wide studies over the past two decades has identified specific human leukocyte antigen (HLA) types that predispose specific populations to SJS/TEN. One of the earlier phenotypes identified was the HLA-B 15:02 allele and the increased risk for SJS/TEN in a Han Chinese population when administered carbamazepine. Genome-wide studies also found an association between HLA-A 31:01 allele and carbamazepine in Europeans and Japanese populations [28,29]. However, a subsequent multinational study could not validate this finding, and reported only a weak association for SJS/TEN carbamazepine [30].

In addition to carbamazepine, allopurinol-induced SJS/TEN has been linked to the HLA-B 58:01 allele in both Asian and European populations [31,32]. New susceptibility regions beyond the HLA subtypes have also recently been reported, including IKZFI as a susceptibility gene for SJS/TEN in Japanese, Korean, and Indian patients [33].

The utility of pharmacogenomics testing is also now translating into screening recommendations for Han Chinese populations. This followed a successful pilot of genome testing throughout Taiwan for a Han Chinese population. In this study, patients were screened for the HLA-B 15:02 allele before the initiation of carbamazepine treatment, and it was found that withholding carbamazepine from HLA-B 15:02-positive subjects reduced the incidence of SJS–TEN [34]. Based on these favourable reports, the US Food and Drug Administration recommended HLA-B 15:02 screening in Han Chinese patients prior to the administration of carbamazepine.

## 6. Clinical Presentation

**Figure 1:**
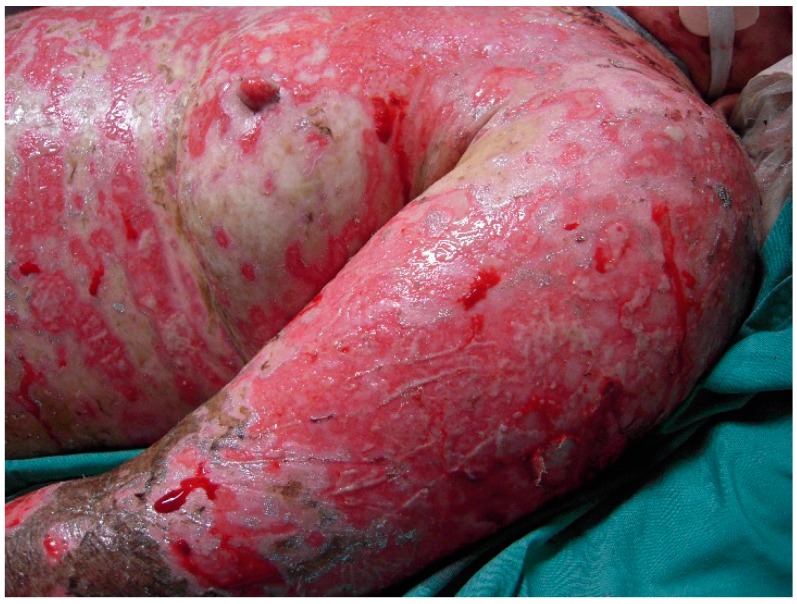
Toxic epidermal necrolysis (TEN). Clinical photograph of patient with widespread denudation of the epidermis in sheets.

**Figure 2:**
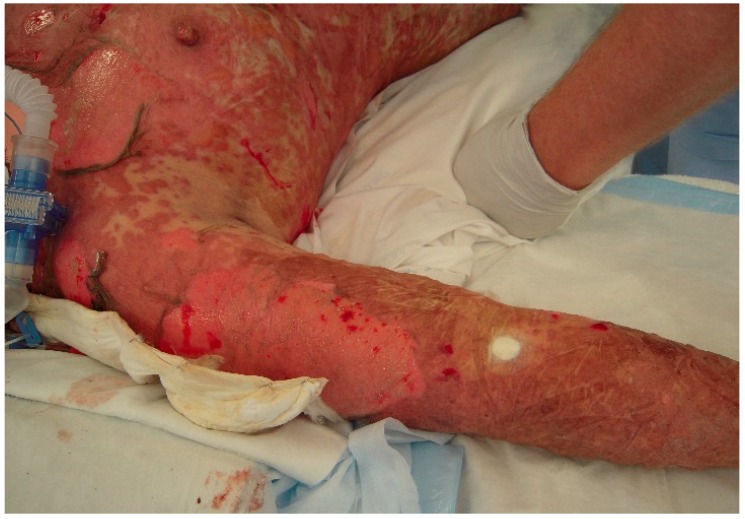
Patient with denudation of the epidermis on the arms and torso resembling wet cigar paper.

The initial signs and symptoms of TEN is a prodrome of malaise, fever, and respiratory tract infection. The earliest lesions are atypical targets or purpuric macules on the face and upper torso and limbs; these increase in size and coalesce as the disease disseminates rapidly into vesicles or fluid blisters (Figure 1 and Figure 2). Detachable skin is demonstrated, and gentle pressure causes detachment of epidermis from dermis (known as a positive Nikolsky sign).

**Figure 3:**
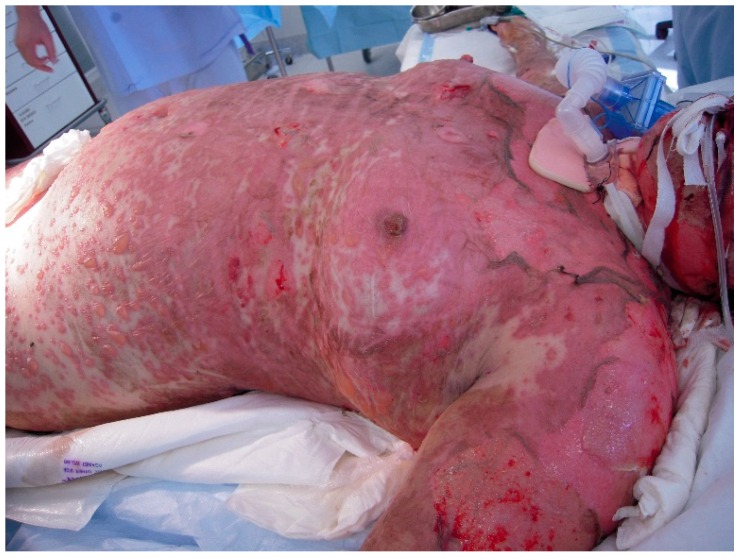
Extensive blisters and erosions affecting >30% body surface areas and airway involvement requiring intubation.

Mucosal involvement occurs both internally and externally in TEN [35]. Airway compromise from disruption to mucosal surfaces is an important feature to be aware of, and symptoms of dyspnoea and hypoxia may herald insidious pulmonary decline not necessarily seen on a chest radiograph (Figure 3) [36].

Care must also be taken to protect the ocular and urogenital mucosa with judicious use of catheters and careful handling due to the sloughing of skin surfaces that can occur. Ocular complications include conjunctivitis, corneal ulceration, and anterior uveitis, and early involvement of ophthalmology is useful. Table 1 provides a summary of both the short- and long-term extra-cutaneous complications that occur in these patients [7]. Multi-disciplinary care within a high dependency or intensive care setting is required for these patients during the acute illness.

The barrier function of the skin is completely compromised in TEN, and as a result, infection and sepsis leading to multi-organ failure is the most common cause of death in these patients [37]. These patients require multidisciplinary care in an intensive care setting due to the multi-organ and life threatening nature of the disease.

## 7. Classification of Severity

The prognosis of TEN can be rapidly assessed in the early stages of the disease via a severity-of-illness score for toxic epidermal necrolysis (SCORTEN). SCORTEN is a validated assessment tool based on seven easily-obtained clinical and laboratory factors, as shown in Table 2 [1]. The mortality rate increases from 3.2% with a score of 0–1 to >90% with a score of 5 or more.

## 8. Management

An important principal of the management of TEN is early diagnosis and identification then cessation of the culprit drug. Initial management and supportive care in a specialised burns unit or intensive care setting is important due to the sequelae of acute skin failure: anaemia, leucopoenia, kidney and liver impairment, and sepsis. The type and mode of administration of analgesia is an important consideration due to the fragility of the skin. Patient-controlled analgesia (PCA) may not be practical due to sloughing of skin on hands, and oral administration may be contraindicated due to oral mucosal disease.

The gaps in the knowledge of the mechanism of TEN and the lack of randomised control trials of pharmacological interventions means that systemic treatment of TEN remains controversial, and the use of adjunctive therapies is a matter of clinical judgment. The most recent European guidelines for TEN do not consider any of the data currently available to be of sufficient quality or consistency to make any recommendations for the use of active treatments [7]. Historically, the systemic therapies used in TEN have included corticosteroids, intravenous immunoglobulin, and cyclosporine. More recently, anti-TNF inhibitors have been suggested as a possible therapeutic agent with limited success, and were found to be detrimental in the case of thalidomide. Therefore, there is an apparent need for future research in the systemic treatment for TEN. The following is a summary of the current systemic treatments that are used, despite the lack of data on their efficacy.

### 8.1. Systemic Corticosteroids

Corticosteroids have historically been used in the management of TEN at high doses, despite the risk of infection and the paucity of quality studies to justify its use. Two small case series have reported decreased anticipated mortality rates with high dose corticosteroids dexamethasone (100 mg IV for 3 days) [38] and methylprednisone (1000 mg IV for 3 days) [39]. In the absence of larger-powered quality data, the use of corticosteroids has not been recommended in recent UK guidelines [7].

### 8.2. Intravenous Immune Globulin (IVIg)

IVIg was proposed as a potential therapeutic agent for TEN based on its ability to significantly block keratinocyte apoptosis by inhibiting the Fas receptor (a mediator for keratinocyte death) [23]. Based on this sole aspect of the mechanism of TEN, IVIg became an accepted treatment for TEN. However, the studies for IVIg have been contradictory, with some showing improved mortality benefit and others showing no mortality benefit. A comprehensive meta-analysis on the use of high dose IVIg reported there was no supporting evidence for a mortality benefit in adults, and IVIg dose did not correlate with mortality [40]. The lack of mortality benefit for patients treated with IVIg has subsequently been confirmed in two studies. The first study by Firoz [41] found no improved survival in a cohort of 23 patients receiving IVIg versus supportive care alone. A second study by Lee [42] in 2013 included a retrospective analysis of 64 patients with SJS/TEN overlap treated with IVIg, and showed no improved mortality from predicted outcome based on SCORTEN. Therefore, while IVIg has previously been an accepted therapeutic agent in TEN, its efficacy is becoming more questionable. Despite these studies, the current European guidelines on the use of IVIg in dermatology have suggested that it should be used early in the disease process in the absence of an alternative evidence-based therapeutic alternative [43], given that the potential benefits of high-dose IVIg outweigh the risks of the medication and the disease’s natural course.

### 8.3. Cyclosporine

The trend in the literature away from IVIg continued in 2014, when a retrospective review on the treatment of TEN with IVIg versus cyclosporine found cyclosporine to have greater mortality benefit [44]. Cyclopsorine has been used due to its theoretical benefit in TEN with its function of inhibiting lymphocytes and its potential anti-apoptotic effects [26]. Based on the favourable outcomes of a French open label trial of cyclosporine for SJS/TEN, a generally acceptable dose of cyclosporine is 3 mg/kg over 7 days followed by a tapering dose [45].

### 8.4. TNF-α Antagonists (Infliximab, Entanercept, Thalidomide)

Recently, there has been an interest in the role of TNF-α inhibitors preventing apoptosis in TEN [16]. Two cases have been reported that showed rapid resolution of skin lesions in TEN after systemic anti-TNF-α therapy with infliximab (5 mg/kg as single-shot therapy) [46]. Entanercept is another TNF-α inhibitor that had promising results in a series of 10 patients with SJS/TEN who were given single 50 mg subcutaneous dose with rapid re-epithelialisation and no deaths despite a SCORTEN-predicted mortality of 50% [47]. These cases support the theory that TNF-α produced by keratinocytes (as well as monocytes–macrophages) is significantly involved in the tissue damage [48].

Thalidomide is another potent inhibitor of TNF-α that was convincingly found to be detrimental to patients for the treatment of TEN in the only randomised control trial performed for the disease [49]. There was a convincing finding that thalidomide was detrimental in TEN, with far greater deaths in the group that received thalidomide versus the control. It is hypothesised this is due to a paradoxical enhancement of TNF-α production due to its co-stimulatory T cell effects.

### 8.5. Granulocyte Colony Stimulating Factor (G-CSF)

G-CSF was reported to have beneficial clinical results in two cases of severe TEN affecting over 80% body surface area (BSA) and concurrent neutropenia [50]. In both cases, the patients made full recovery. It is hypothesised the favourable outcomes were in part due to the boost in bioregeneration or accelerated re-epithelialisation from the granulocyte colony stimulating factor (G-CSF).

## 9. Conclusions

TEN is a life-threatening immune-mediated drug reaction associated with high morbidity and mortality rates. The exact mechanisms of the disease remain unknown, but granulysin is generally accepted as the primary mediator of the CTL driven keratinocyte death. There has been substantial development in the prevention of SJS/TEN through the increased knowledge of the genetic predisposition to the SJS/TEN. Early diagnosis and identification of the culprit drug is the mainstay of treatment of the disease. Pharmacological interventions remain contentious with the absence of quality, randomised study. However, there is a trend away from the traditionally accepted role of IVIg towards the use of anti-TNF-α antagonists. However, there needs to be further more robust studies into these systemic therapies. The gold-standard remains early diagnosis, removal of culprit drug, and a focus on supportive care.

## Figures and Tables

**Table 1:** Management of extra-cutaneous complications of toxic epidermal necrolysis.

System	Short Term Complications	Daily Specialist Review during Admission	Management	Long Term Complications
Ocular	Ocular surface damage, infection	Yes	Topical lubricant (non-preserved hyaluronate), corticosteroid drops (non-preserved dexamethasone 0.1%), topical antibiotic prophylaxis (moxifloxacin)	Corneal and conjunctival ulceration and scarring, dry eye, entropion, visual impairment, blindness
Oral	Infection, pain	Yes	White soft paraffin ointment to oral mucosa, warm saline mouthwashes, corticosteroid mouthwash (betamethasone sodium phosphate), antiseptic mouth rinse	Scarring may cause food trapping, limitation of oral mobility
Urogenital	Infection, erosions, pain	Yes	White soft paraffin ointment to the urogenital skin, potent topical corticosteroid ointment, silicone dressing to eroded surfaces	Adhesions: vaginal introital, dyspareunia, scarring, pigment changes
Respiratory	Hypoxia, airway compromise, infection, bronchial erosions	Intensive Care Unit (ICU) or High Dependency Unit (HDU) admission	Fibre optic bronchoscopy, ICU or HDU monitoring	Bronchiolitis obliterans causing severe airway obstruction

**Table 2:** SCORTEN SCORE (severity-of-illness score for toxic epidermal necrolysis)—prognostic factors for toxic epidermal necrolysis (TEN) provide a score of 0 or 1.

Risk Factor	Score 0	Score 1
Age	<40 years	>40 years
Tachycardia	<120 beats per min	>120 beats per min
Malignancy	No	Yes
Body surface area detached	<10%	>10%
Serum urea	<10 mmol/L	>10 mmol/L
Serum glucose	<14 mmol/L	>14 mmol/L
Serum bicarbonate	>20 mmol/L	<20 mmol/L
